# Three-Dimensional Printing and Electrospinning Dual-Scale Polycaprolactone Scaffolds with Low-Density and Oriented Fibers to Promote Cell Alignment

**DOI:** 10.1089/3dp.2019.0091

**Published:** 2020-06-05

**Authors:** Cian Vyas, Gokhan Ates, Enes Aslan, Jack Hart, Boyang Huang, Paulo Bartolo

**Affiliations:** ^1^ Department of Mechanical, Aerospace, and Civil Engineering and University of Manchester, Manchester, United Kingdom.; ^2^ Department of Physics and Astronomy, University of Manchester, Manchester, United Kingdom.

**Keywords:** 3D printing, biomaterials, electrospinning, scaffolds, tissue engineering

## Abstract

Complex and hierarchically functionalized scaffolds composed of micro- and nanoscale structures are a key goal in tissue engineering. The combination of three-dimensional (3D) printing and electrospinning enables the fabrication of these multiscale structures. This study presents a polycaprolactone 3D-printed and electrospun scaffold with multiple mesh layers and fiber densities. The results show successful fabrication of a dual-scale scaffold with the 3D-printed scaffold acting as a gap collector with the printed microfibers as the electrodes and the pores a series of insulating gaps resulting in aligned nanofibers. The electrospun fibers are highly aligned perpendicular to the direction of the printed fiber and form aligned meshes within the pores of the scaffold. Mechanical testing showed no significant difference between the number of mesh layers whereas the hydrophobicity of the scaffold increased with increasing fiber density. Biological results indicate that increasing the number of mesh layers improves cell proliferation, migration, and adhesion. The aligned nanofibers within the microscale pores allowed enhanced cell bridging and cell alignment that was not observed in the 3D-printed only scaffold. These results demonstrate a facile method of incorporating low-density and aligned fibers within a 3D-printed scaffold that is a promising development in multiscale hierarchical scaffolds where alignment of cells can be desirable.

**Figure:**
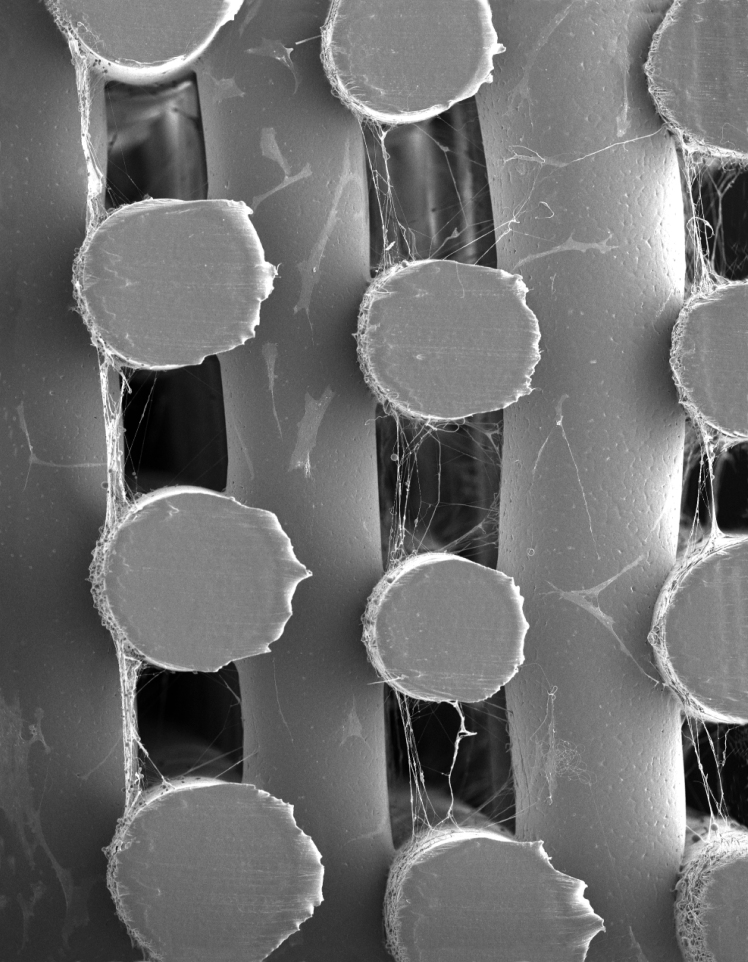


## Introduction

Additive manufacturing is enabling the development of complex, multimaterial, functionally graded, and patient-specific structures for tissue engineering applications.^1^ Extrusion-based three-dimensional (3D) printing is a commonly utilized technique for tissue engineering, allowing the fabrication of structures consisting of both hard and soft materials. These structures or scaffolds allow cell attachment, proliferation, and the generation of new tissues. The precise deposition of biomaterials that can contain biological materials such as cells and growth factors is a promising development within tissue engineering and allows the native structure of tissues and organs to be more accurately mimicked.^2^

However, a major limitation of extrusion-based 3D printing is the resolution of the features fabricated are above a few microns. Key features in the extracellular matrix (ECM) that surround cells and tissues are submicron. This requires specific designing to mimic or incorporate submicron features within a tissue-engineered scaffold to modulate cell behavior.^3–5^ Currently, there is difficulty incorporating distinct scale lengths within the same structure that are specifically engineered, limiting the biological suitability of the structure.

Electrospinning provides an opportunity to introduce nanoscale fibers, mimicking the ECM, into the 3D-printed structure.^6–9^ The 3D network composed of both printed and electrospun micro- and nanofibers is a promising approach owing to the creation of a structure that is mechanically stable while maintaining interconnectivity, high porosity, a large surface area to volume ratio, and providing nanoscale 3D features for cell attachment. This nanoscale topography provides a large surface area for cell attachment and bridging between microscale fibers that can form a microenvironment enhancing cell interaction and mobility. Furthermore, cell behavior can be modulated by the topographical features of the fibrous mesh as fiber diameter, distribution, alignment, and porosity can be controlled.^10–13^ These features facilitate specific cell adhesion, morphology, migration, proliferation, polarity, integrin clustering, and differentiation through mechanotransduction signaling pathways.

Typically, previous studies incorporating 3D printing and electrospinning have spun only onto the top surface of the scaffold, directly placing meshes into the printed structure, infiltrating dispersed nanofibers into the scaffold, or directly combining printing and electrospinning.^6–9^ However, these studies produced highly dense meshes limiting cell migration and infiltration, which is a common issue with electrospinning.^14^ In addition, these highly dense meshes only provide a 2D architecture for cell attachment onto the mesh mediated through adhesion molecules rather than the 3D attachment in native tissues. These highly dense meshes can limit the cell's tissue-specific remodeling of their surroundings and the secretion of the desired ECM that can be crucial in the development of functional tissue.

Furthermore, the fibers are randomly distributed as is typical with electrospinning and thus topographically induced cell behavioral cues such as alignment were not observed. Electrospinning oriented fibers can be achieved through a number of techniques such as using a rotating mandrel collector, conductive electrodes separated by an insulating gap, a patterned collector, and near-field electrospinning.^15–17^ The ECM in most tissues has an anisotropic architecture, thus the fabrication of aligned fibers is key to mimicking the native structure and has a significant effect on cell behavior and tissue regeneration.^15,18–21^

The combination of 3D printing and electrospinning utilizing a low-density mesh to facilitate cell migration and aligned fibers to modulate cell behavior is a promising approach to improve tissue-engineered scaffolds. In this study, we developed a dual-scale scaffold by coupling 3D-printed microfibers directly with electrospun nanofibers throughout the scaffold. Our aim is to produce highly aligned electrospun fibers within a 3D-printed scaffold to modulate cell behavior. The printed fibers will act as a gap collector to orient the electrospun fibers. Electrospun mesh density (as a function of electrospinning time), number of meshes dispersed through the scaffold, and fiber orientation were investigated morphologically and biologically. Aligned nanofibers were produced within the 3D-printed pores due to the printed microfibers acting as both a gap and patterned collector. The dual-scale scaffolds supported higher cell attachment and proliferation with the aligned fibers enabling cell alignment and bridging between printed fibers.

## Materials and Methods

### Scaffold fabrication

**FIG. 1.**
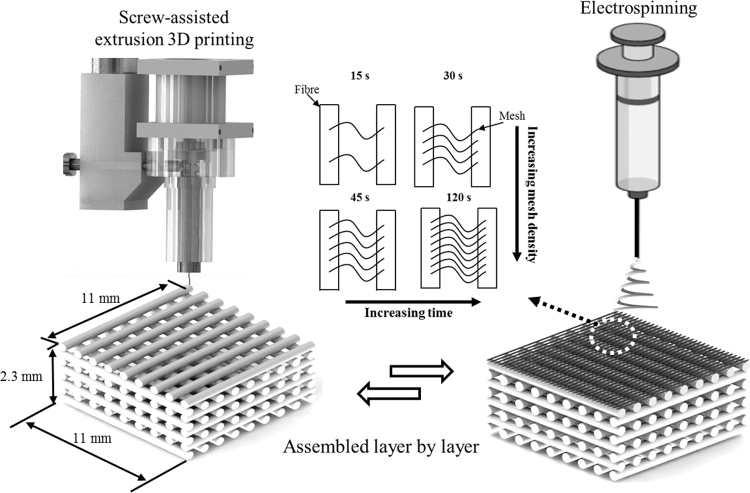
Schematic representation of the 3D printing and electrospinning fabrication process of a dual-scale scaffold. 3D, three-dimensional.

A screw-assisted extrusion 3D printer (3D Discovery, RegenHU, Switzerland) and electrospinning system (Spraybase, Ireland) were employed to create polycaprolactone (PCL, Mw 50,000; Perstorp, United Kingdom) dual-scale scaffolds (Fig. 1). 3D-printed scaffolds were designed with a 0°/90° lay down, 300 μm pore size and fiber diameter, and 230 μm layer height. The pore size of 300 μm was selected as studies have shown this to be within a range to achieve optimal cell proliferation, tissue ingrowth and neovascularization *in vivo* for bone tissue engineering applications.^22,23^ Processing parameters used were a 0.33 mm inner diameter (ID) nozzle, 90°C melt temperature, 12 mm/s deposition speed, and screw rate of 7.5 rpm. The printed scaffolds had designed dimensions of 11 (*W*) × 11 (*L*) × 2.3 mm (*H*), equivalent to 10 printed layers, and were printed onto circular steel disks, 15 (*D*) × 3 (*H*) mm, which allowed 15 scaffolds to be printed simultaneously on the with build platform. The steel disks also allowed the platform to be conductive for subsequent electrospinning.

Electrospun fibers were produced using a 24% w/v PCL in acetic acid solution, flow rate of 0.75 mL/h, 15 cm needle collector distance (kept constant), 14 kV charge, and a 1 mm ID needle. The polymer solution was prepared by dissolving PCL in glacial acetic acid while being stirred at 40°C for 24 h. Fiber mesh densities of 15, 30, 45, and 120 s of electrospinning time were considered. Dual-scale scaffolds with 1, 3, and 5 meshes evenly spaced throughout the scaffold were produced by electrospinning directly onto the scaffold at the appropriate layer during printing. During the 3D printing process, the G-code was preprogrammed to pause at a specific layer (1 mesh: layer 5; 3 mesh: layers 3, 6, and 9; 5 mesh: layers 1, 3, 5, 7, and 9) to allow electrospinning before the printing was resumed.

### Morphological characterization

Scaffold morphology was assessed using scanning electron microscopy (SEM; Hitachi S-3000N, Japan). Samples were sputter coated (108 auto; Cressington, United Kingdom) with platinum for 60 s (∼10 nm thickness). Images were analyzed using Fiji and relevant dimensions were evaluated.^24^

Fiber orientation was identified using the biopolymer network software SOAX, which utilizes stretching open active contour models to identify fibers in the SEM images based on the ridge pixel intensity (gray-scale value) compared with the background.^25^ The fiber orientation with respect to the *x*-axis of each image was calculated using a vector analysis of the opposing ends of identified fibers. The same protocol was followed to identify cell orientation; however, confocal images of cells were used in place of SEM images of fibers.

### Mechanical testing

Compression tests were performed on the scaffolds (*n* = 4) using a rate of 0.5 mm/min up to a strain limit of 45% using a universal testing machine (Instron 4507, USA) equipped with a 2 kN load cell.

### Wettability

Wettability as a function of electrospun fiber mesh density (0, 15, 30, 45, 120 s) on the top layer of the 3D-printed scaffold was assessed. The water-in-air contact angle (KSV CAM 200; KSV Instruments, Finland) of the scaffolds (*n* = 3) was measured.

### Biological assessment

Human adipose-derived stem cells (STEMPRO; Invitrogen, USA) were used for biological characterization. Cells were cultured in MesenPRO basal medium, 2% (v/v) growth supplement, 1% (v/v) penicillin/streptomycin, and 1% (v/v) glutamine (Invitrogen) within a cell culture incubator. Scaffolds were sterilized using 80% ethanol, washed with phosphate-buffered saline (PBS) solution, and dried overnight. Cells were trypsinized and seeded at passage 11 onto each scaffold at density of 5 × 10^4^ cells in 150 μL of medium followed by 2 h of incubation to allow cell attachment before addition of medium. Subsequently the medium was changed every 2 days.

Cell proliferation was assessed using the Alamar blue assay (Sigma-Aldrich, United Kingdom) at days 1, 3, 7, and 14. Samples (*n* = 4) were transferred to a new 24-well plate and a 0.01% w/v Alamar blue solution was added, final concentration 0.001% w/v, and incubated for 4 h. The fluorescence signal was read (540 nm excitation/590 nm emission) using a microplate reader (Infinite 200; Tecan, Switzerland). Samples were washed with PBS and medium was added.

Cell attachment, morphology, and distribution were observed using SEM and confocal laser scanning microscopy (CLSM; Lecia TCS-SP5; Lecia Microsystems, Germany). Scaffolds were washed with PBS and separately fixed in 2.5% glutaraldehyde (Sigma-Aldrich) for 1 h and 10% formalin for 30 min for SEM and CLSM, respectively.

Samples for SEM were dehydrated in sequentially increasing concentration of ethanol (50–100%) for 15 min each, twice at 100%, 15 min in a 50:50 solution of hexamethyldisilazane (Sigma-Aldrich) and ethanol, and then 100% hexamethyldisilazane. Samples were coated in platinum.

CLSM imaging was obtained by using 4′,6-diamidino-2-phenylindole (DAPI) and Alexa Fluor 488-conjugated phalloidin (Thermo Fisher Scientific, United Kingdom) to stain the nucleus and actin, respectively. Samples were permeabilized using 0.1% Triton X-100 (Sigma-Aldrich) for 10 min and rinsed with PBS. Scaffolds were stained with a 1:400 dilution of Alexa Fluor 488-conjugated phalloidin for 45 min then with 1 μg/mL DAPI solution for 5 min.

### Statistical analysis

One-way analysis of variance with Tukey *post hoc* test (GraphPad Prism 8; GraphPad Software, USA) was performed and results are presented as the mean ± standard deviation. Significant differences were considered at *p < 0.05.

## Results and Discussion

**FIG. 2.**
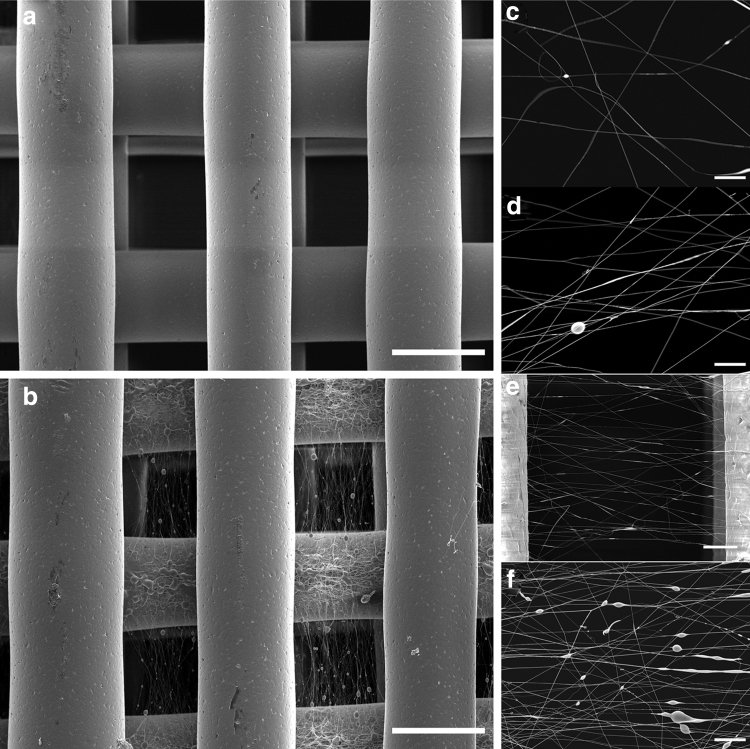
SEM images of the **(a)** 3D-printed only scaffold and **(b)** dual-scale scaffold with electrospun (45 s) nanofibers (scale bar = 300 μm). Electrospun mesh density as function of time **(c)** 15 s, **(d)** 30 s, **(e)** 45 s, and **(f)** 120 s [scale bar = 20 μm, **(e)** 50 μm]. SEM, scanning electron microscopy.

Dual-scale scaffolds were successfully fabricated with a regular printed structure and the 3D-printed fibers having a circular geometry and diameter of 287.2 ± 27.5 μm with a pore size of 299.2 ± 18.3 μm (Fig. 2). The electrospun fibers were spun onto the scaffolds at specific layers and had a fiber diameter of 820 ± 56 nm. Beads can be observed on electrospun fibers on the printed fibers and within the pores. This may be due to the dissipation of charge as the scaffold thickness increases, the poor conductive properties of the 3D-printed PCL scaffold, and the use of acetic acid as a solvent all of which can result in instability in the charged polymer jet. Fiber alignment is observed on meshes spun for 30 s or more within the printed microfiber pores (Fig. 2d).

**FIG. 3.**
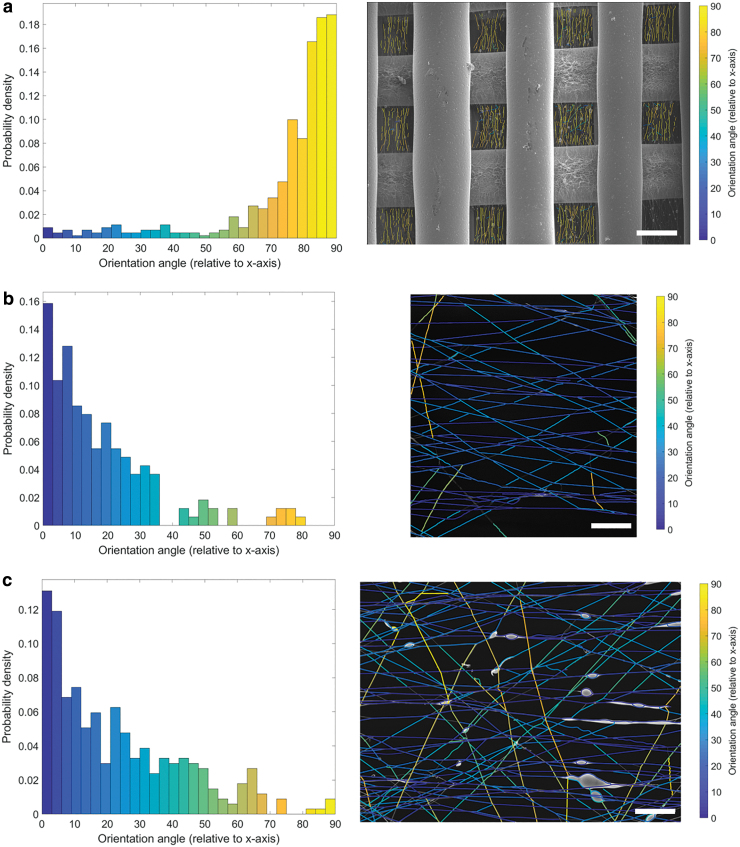
Fiber orientation analysis of SEM images. **(a)** Low magnification image showing aligned electrospun fibers (45 s) within all the pores of the printed scaffold (scale bar = 300 μm). Probability density of the orientation angle (relative to *x*-axis) shows a distinct distribution for angles between 75° and 90°. Higher magnification images **(b)** 45 s (scale bar = 50 μm) and **(c)** 120 s (scale bar = 20 μm) show aligned fibers with the orientation angle distributed toward 0°. Color images are available online.

Fiber orientation analysis demonstrates a clear preference for electrospun fibers to be aligned perpendicular to the direction of the printed fibers (Fig. 3). The electrospun fibers act as bridges between the printed fibers. The aligned fibers are present and homogeneously distributed throughout and within all the 3D-printed pores (Fig. 3a). Furthermore, the electrospun fibers collected on the printed fibers themselves are oriented although not as clearly as the fibers within the pores. These results can be attributed to the electrostatic interactions between the spinning nanofibers and the polarization induced in the microfibers that acts as the fiber collector. The printed microfibers become a series of gap collectors with the microfibers acting as electrodes and the pores (air) as the insulator. This electrode–insulator collector introduces a preferential direction in charge and the electrostatic interactions become direction dependent resulting in the electrospun fibers to be stretched and collected in a uniaxially specific direction.^26^ The scaffold also functions as a patterned collector as well, which may also influence the directionality of the electrospun fibers.^27^ Furthermore, the geometry of the printed fibers also only allows collection of electrospun fibers in a specific direction. Nanofibers that are collected parallel to the direction of the printed fiber are either deposited on top of the printed fiber itself or are aligned within the pore of the previous layer, which in this case is perpendicular to the current layer. The aligned fibers in this study agree with the results of a scaffold developed by Mota *et al.* in which an electrospun mesh was spun onto the top layer of the scaffold and oriented fibers were observed.^8^

**FIG. 4.**
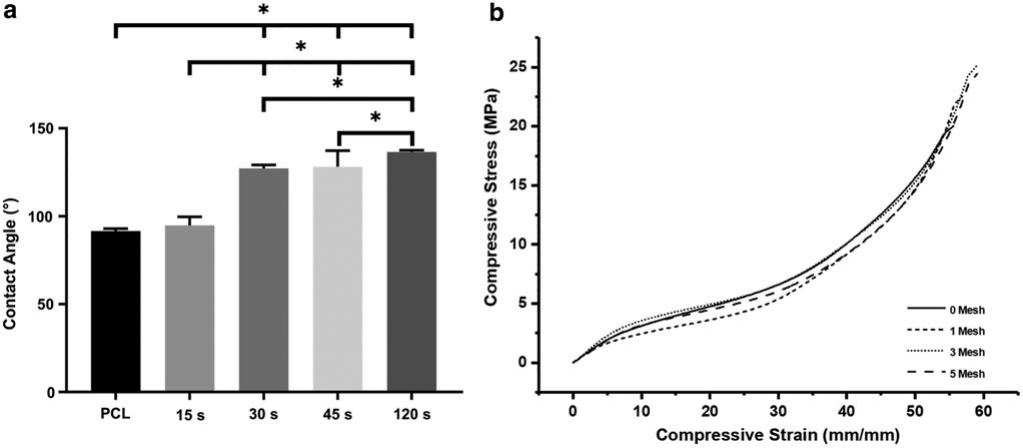
**(a)** Wettability of the dual-scale scaffolds as a function of electrospinning time (*p < 0.05). **(b)** Representative compressive stress–strain curves of the scaffolds.

The influence of mesh density on wettability was observed with increasing collecting time, resulting in a significant increase in the hydrophobicity of the surface (Fig. 4a). PCL is a hydrophobic polymer and thus the decrease in wettability with increasing spinning time can be used as a guide to determine the mesh density. The mesh density was also qualitatively inspected to assess the suitability for cell culture studies according to fiber spacing and porosity. Meshes of 45 s were considered for further biological studies as the meshes with higher spinning times presented relatively dense structures that may hinder cell migration through the scaffold. Whereas lower spinning times produced meshes with high porosity, potentially minimizing cell attachment.

The compressive mechanical properties of the dual-scale scaffolds with 1, 3, or 5 meshes were assessed (Fig. 4b and Table 1). No significant differences were observed in mechanical properties between sample types with all scaffolds behaving with a typical cellular solid stress–strain profile.^28^

**Table 1.** Compressive Modulus, Within the Linear Elastic Region, of the Dual-Scale Scaffolds as a Function of the Number of Electrospun Meshes

Meshes	Compressive modulus (MPa)
0	35.18 ± 1.76
1	37.95 ± 0.95
3	35.52 ± 2.62
5	29.55 ± 1.14

The quantity of electrospun fibers may not be at a critical value to impose measurable differences in the mechanical properties. However, these results are similar to the dual-scale system investigated by Kim *et al.* in which the addition of electrospun fibers did not increase the compressive properties of the scaffold.^6^ Further investigation of the role mesh density has on mechanical properties is required. In particular, the presence of electrospun fibers within the pores may alter the tensile and shear properties of the scaffold and requires specific testing to evaluate these properties. For example, fluid flow within the scaffold may be different depending on location, printed, or electrospun fiber, thus the shear forces experienced by attached cells will be distinct. This extends to the tensile and compressive forces experienced as two separate mechanical regimes are imposed on the printed and electrospun fibers of the dual-scale scaffold. In addition, the electrospun fiber deposited along the printed fiber may influence the weld, interlayer binding interface, between the 3D-printed fibers. As 3D-printed structures typically exhibit anisotropic mechanical properties in the build direction, changes in weld quality may further influence this.^29^ The preliminary mechanical assessment warrants further experimental investigation including shear, tensile, and cyclic loading regimes. The currently designed and fabricated dual-scale scaffold needs specific optimization in material composition and architecture to enable compliance with the complex mechanical environment required in tissue engineering applications.

**FIG. 5.**
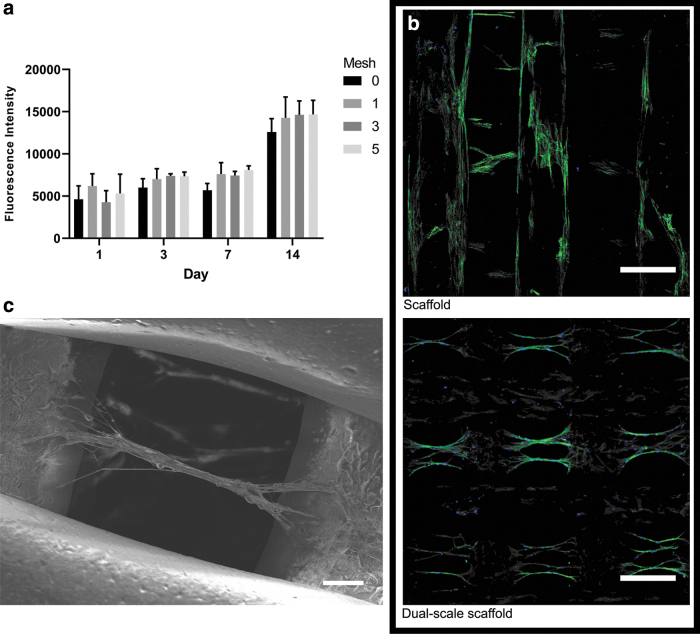
**(a)** Alamar blue results for cell proliferation up to 14 days. **(b)** CLSM images of cell seeded 3D-printed only and dual-scale scaffolds with actin (*green*) and cell nuclei (*blue*) staining (scale bar = 300 μm). **(c)** SEM image of cell alignment and bridging in a dual-scale scaffold (scale bar = 50 μm). CLSM, confocal laser scanning microscopy. Color images are available online.

Biological analysis of the dual-scale scaffolds demonstrates a trend for increasing cell proliferation on dual-scale scaffolds, especially with higher number of mesh layers (Fig. 5a). However, there is no significant difference between sample types as the overall surface area within the dual-scale scaffolds will be relatively similar regardless of the number of electrospun meshes due to their low density. The bridging between the printed microfibers provided by the electrospun fibers allows an appropriate environment for cell adhesion, proliferation, and migration throughout the entirety of the scaffold and is responsible for the trend in higher cell proliferation in electrospun mesh containing samples.

**FIG. 6.**
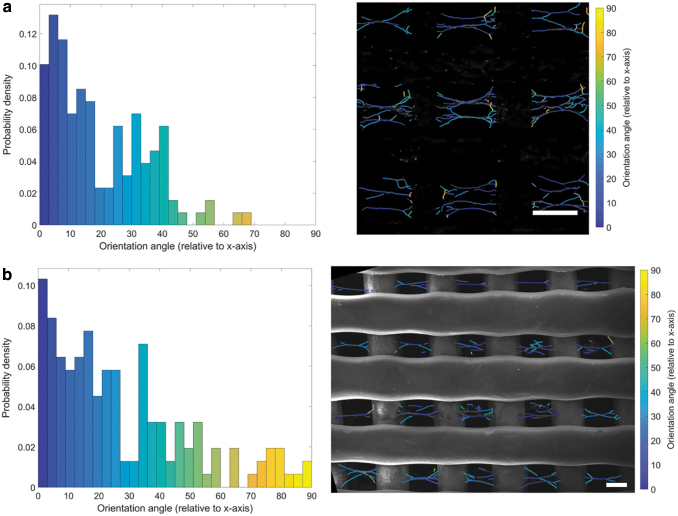
Cell orientation analysis. **(a)** Confocal (scale bar = 300 μm) and **(b)** SEM (scale bar = 150 μm) images of the cells cultured on the dual-scale scaffold (45 s) show aligned cell bodies with a high probability density at 0° and another distinct distribution at ∼40°. Color images are available online.

Cell morphology was observed using CLSM on day 14 (Fig. 5b). Cell attachment is primarily on the 3D-printed microfibers on day 1; however, by day 14, a clear difference in cell migration and morphology is observable between the printed only and dual-scale scaffold. Extensive cell proliferation is observed throughout both types of scaffold by day 14. The dual-scale scaffolds exhibit cell–nanofiber bundles with cell alignment corresponding to the orientation of the aligned nanofibers. This phenomenon was confirmed additionally through SEM (Fig. 5c). Cell–fiber orientation analysis demonstrates highly aligned cell bodies within the pores and a second distribution population (∼40°) corresponding to the original attachment points of the electrospun fibers and now angled as a branching point for the main fiber–cell bundle (Fig. 6). Clusters of cells are observed in the individual bundles and multiple cell–nanofibers bundles are bridging between the printed fibers. Cell morphology is considerably more elongated than the cells attached on the printed microfibers. This behavior is unique to the dual-scale scaffold with the 3D-printed only scaffold showing typical cell–microfiber interactions with cells spreading across the printed fibers. The cells can also remodel the electrospun fibers as can be observed in the difference in the mesh density and electrospun fibers visible before and after cell seeding (Figs. 2 and 5). The cells seem to physically pull and move the nanofibers that results in the change of cell morphology observed between the nanofibers and the printed microfibers. The electrospun nanofibers that were distributed across the pore are now, after cell seeding, arranged into fiber–cell bundles in the middle of the pore that branches off and connects with the printed fiber when in proximity to the microfiber (Fig. 6a). A higher density mesh would resist the deformation by the cells resulting in a different cell morphology. Subsequently, further investigation is required to determine how altering the density of the suspended mesh between the two printed fibers can affect the mechanical properties of the mesh and thus how the cells sense the surface resulting in changes to mechanotransduction pathways, cell morphology, and behavior.

The presence of aligned nanofibers enables the elongation, guidance, and alignment of cells while facilitating the colonization of the scaffold by providing bridging between printed microfibers. Topographical alignment is a key factor in cell morphology, adhesion, and differentiation. For example, aligned fibers have been demonstrated to promote osteogenic differentiation;^30–34^ enhance axon guidance, neurite outgrowth, and guiding Schwann cells toward a promyelinating state;^35–38^ increase myogenic elongation and differentiation;^39–41^improve regeneration and differentiation toward tendon and ligament lineages;^42–44^ cartilage regeneration;^45^ and enhancing the contractility, organization, and electrical transmission in cardiac tissues.^46–50^ Subsequently, the generation of aligned nanofibers in a simple methodology, as described in this study, is of great interest in tissue engineering applications.

## Conclusions

A dual-scale scaffold composed of 3D-printed and electrospun PCL fibers was successfully fabricated providing both micro- and nanoscale features. Aligned electrospun nanofibers were produced within the porous structure of the 3D-printed scaffold, which is highly relevant in tissue engineering applications to modulate cell behavior. A facile method of incorporating aligned and low-density electrospun meshes into a 3D-printed scaffold was demonstrated as the printed scaffold acted as a combined gap and patterned collector for the charged jet of the polymer solution. Biological assessment demonstrated that cell proliferation increased in the dual-scale scaffolds and aligned cells with an elongated morphology were observed on the mesh in the pores of the printed microfibers. Further investigation is required to understand how the conductivity of the material influences fiber formation and alignment potentially through the incorporation of conductive fillers such as graphene or the use of conductive polymers. The electrical charge distribution can also be altered by changing the printed scaffold geometry (e.g., hexagonal and triangular) and incorporating both conductive and insulating regions within the structure to influence fiber alignment. This study is a promising development in the fabrication of multiscale scaffolds that better reflect the complexity of native tissue and the ability to engineer specific architectures to control cell behavior.
